# Amino Acids Involved in Polyphosphate Synthesis and Its Mobilization Are Distinct in Polyphosphate Kinase-1 from *Mycobacterium tuberculosis*

**DOI:** 10.1371/journal.pone.0027398

**Published:** 2011-11-14

**Authors:** Payal Mittal, Subramanian Karthikeyan, Pradip K. Chakraborti

**Affiliations:** Council of Scientific and Industrial Research—Institute of Microbial Technology, Sector 39A, Chandigarh, India; University of South Florida College of Medicine, United States of America

## Abstract

**Background:**

In bacteria polyphosphates (poly-P) are involved in cellular metabolism and development especially during stress. The enzyme, principally involved in polyphosphate biosynthesis and its mobilization leading to generation of NTPs, is known as polyphosphate kinase (PPK).

**Principal Findings:**

Among two genes of polyphosphate kinases present in *Mycobacterium tuberculosis*, we cloned and expressed PPK1 in *Escherichia coli* as histidine-tagged protein. This ∼86 kDa protein is capable of autophosphorylation and synthesis of poly-P as well as NTP. Among 22 conserved histidine residues, we found only His-491 is autophosphorylated and crucial for any enzymatic activity. Substitution of His-510 caused mPPK1 protein deficient but not defective in autophosphorylation, thereby contrary to earlier reports negating any role of this residue in the process. However, mutation of His-510 with either Ala or Gln affected ATP or poly-P synthesis depending on the substitution; while such effects were severe with H510A but mild with H510Q. Furthermore, mPPK1 also renders auxiliary nucleotide diphosphate kinase function by synthesizing virtually all NTPs/dNTPs from their cognate NDPs/dNDPs by utilizing poly-P as the phosphate donor albeit with varied efficiency. To assess the influence of other catalytic domain residues of mPPK1 towards its functionality, we designed mutations based on *E. coli* PPK1 crystal structure since it owes 68% amino acid sequence similarity with mPPK1. Interestingly, our results revealed that mutations in mPPK1 affecting poly-P synthesis always affected its ATP synthesizing ability; however, the reverse may not be true.

**Conclusions/Significance:**

We conclude that amino acid residues involved in poly-P and ATP synthesizing activities of mPPK1 are distinct. Considering conserved nature of PPK1, it seems our observations have broader implications and not solely restricted to *M. tuberculosis* only.

## Introduction

The presence of polyphosphates (poly-P) is well-known in all the cells in nature and throughout the phylogeny. It is a linear chain of inorganic phosphates linked by ‘high energy’ phosphoanhydride bonds [1], [2]. The involvement of poly-P in several functions, especially during stress, suggests its importance in cellular metabolism as well as development of an organism [3]–[6]. The enzyme principally involved in polyphosphate biosynthesis is polyphosphate kinase (PPK). Similarly, role of several polyphosphatases in controlling poly-P level has already been reported [7], [8]. Therefore, it is apparent that a homeostasis between the activities of polyphosphate kinases and phosphatases are necessary for maintenance of intra-cellular concentration of poly-P in any organism.

PPK catalyzed reaction is reversible. In addition to poly-P synthesis, known as forward reaction, another important role of this enzyme is to generate ATP, which is termed as reverse reaction [9]–[11]. Thus, this enzyme to some extent behave like the nucleoside diphosphate kinase (NDK), the principal enzyme involved in maintenance of intracellular pool of nucleoside triphosphates or their deoxy derivatives (NTPs/dNTPs). In many bacteria, two different genes *ppk1* and *ppk2* encode PPK enzymes. Interestingly, enzymes encoded by PPK1 and PPK2 have no sequence similarity. PPK1 is predominantly involved in cellular poly-P biosynthesis using terminal phosphate of ATP as the substrate [2], [11], [12]. PPK2, on the other hand, is distinguished from PPK1 by its high poly-P utilization ability for generating ATP and/or GTP in *Pseudomonas aeruginosa* and therefore, often exhibited poly-P dependent NDK activity [13]–[15].

PPK1 is conserved throughout the phylogeny. It is a phospho-protein and forms poly-P through an intermediate in which a phosphate group of ATP is covalently attached to basic histidine residue through an N-P bond [16], [17]. However, the mechanistic detail of PPK1 mediated mobilization of poly-P in synthesizing NTP is not explored in greater detail. The 2.5 Å X-ray structure of PPK1 from *E. coli* has been solved in recent years [18]. *E. coli* PPK1 is comprised of four domains, namely amino terminal (N domain), head (H domain) and two closely spaced carboxy-terminal domains (C1 and C2 domains). While in *E. coli*, the N domain is associated with ATP binding, both C1 and C2 domains are responsible for the catalytic activity of the enzyme. Interestingly, interaction between H and C1 domains of two monomers of *E. coli* PPK1 have been assigned in dimerization of the protein yielding enzymatically active conformation [18].

The role of PPK1 in virulence and pathogenesis is well established in pathogenic bacteria [19], [20]. It is identified as a colonization factor in *Helicobacter pylori* [21]. Its involvement in providing resistance to acid stress in *Salmonella enterica* and in growth of *Burkholderia cepacia* under low-pH conditions is well documented [22], [23]. This enzyme is shown to regulate swimming, swarming and twitching motilities of *P. aeruginosa* [24]. High degree of similarity of PPK1 sequence in many bacteria, including pathogens and its altogether absence in eukaryotes (except *Dictyostelium discoideum*) suggested it as an ideal target for screening of antimicrobial compounds. In this context, we have focused on *Mycobacterium tuberculosis*, the bacteria causing tuberculosis, which is responsible for considerable human mortality throughout the world [25], [26]. Availability of the genome sequences (http://www.jcvi.org) indicated the presence of two genes, *ppk1* and *ppk2*, encoding polyphosphate kinases in different mycobacterial species. In *M. tuberculosis*, association of PPK1 (mPPK1) in stress induced mprAB- sigE- rel signaling pathway [27] and PPK2 (mPPK2) as a modulator of NDK activity have been documented [28].

In this study, we have concentrated mainly on *M. tuberculosis* PPK1 (mPPK1). We demonstrated here that His-491 of mPPK1 is crucial for the functionality of the enzyme. Our results highlighted that mPPK1 is the poly-P synthesizing enzyme. Interestingly, we found that utilizing poly-P as the phosphate donor mPPK1 is also capable of synthesizing virtually all NTPs/dNTPs from their cognate NDPs/dNDPs and thus renders auxiliary NDK activity. PPK2 (mPPK2), on the other hand, was able to synthesize only ATP from poly-P. Furthermore, analysis of different catalytic site residues of the enzyme based on *E. coli* PPK1 structure [18] together with our biochemical experiments indicated that amino acids involved in poly-P and ATP synthesizing activities of the protein are distinct in mPPK1.

## Results

### mPPK1 autophosphorylates at His-491

Cell lysate and Ni-NTA purified cell supernatant prepared from *E. coli* BL21(DE3) cells harboring pET-mPPK1 (*M. tuberculosis ppk1* gene isolated through PCR amplification from genomic DNA and cloned in pET-28c) on resolving in 10% SDS-PAGE yielded 86±0.13 kDa (Mean ± S.D., n = 4) expressed as histidine-tagged protein (Fig. S1A, upper panel), which is very close to its calculated molecular mass (86.3 kDa containing 6 His and 15 additional amino acids from the vector). The authenticity of His-tagged protein was confirmed by Western blotting using anti-His antibody and the polyclonal antiserum raised against mPPK1 in rabbit (Fig. S1A, middle and lower panels respectively). The autophosphorylation ability of the mPPK1 was assessed by incubating different concentrations (25 ng–5 µg) of protein with [γ-^32^P]ATP in the presence of 10 mM Mg^2+^ (or Mn^2+^or other divalent cations) and 40 mM ammonium sulphate at 25°C for 30 min in an *in vitro* kinase assay. Following separation of reaction products on SDS-PAGE, the labeled protein was visualized using a phosphoimaging device as well as in autoradiography. Like other prokaryotes [16], [29], [30], our results revealed that autophosphorylation of mPPK1 increases with increasing amount of protein (Fig. S1B) and divalent cation (Mg^2+^ or Mn^2+^) is necessary for the activity (Fig. S1C).

**Figure 1:**
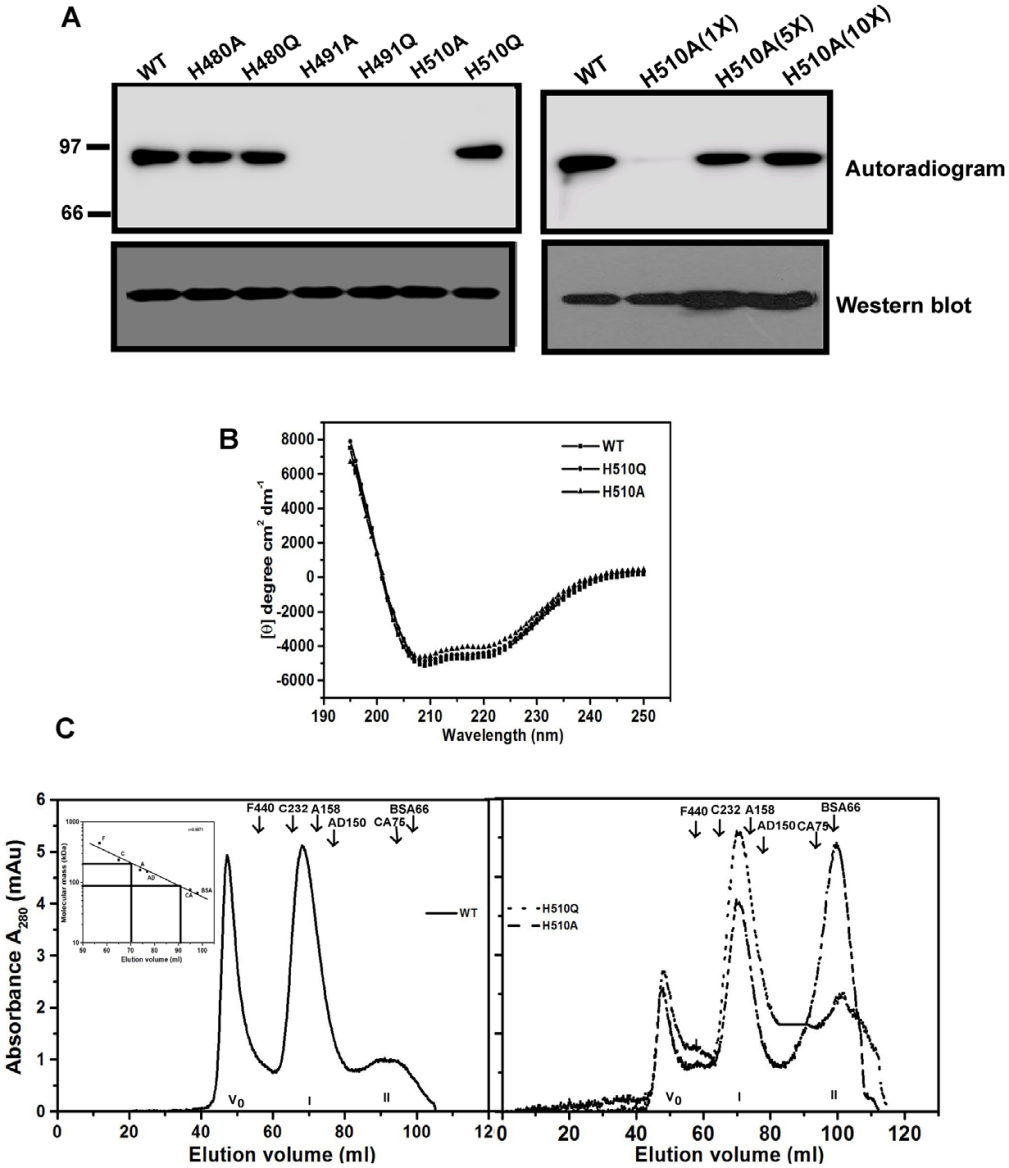
mPPK1 autophosphorylates at His-491 residue. A. Autophosphorylating ability of mPPK1 in response to mutations at conserved histidine residues. Upper left panel: Autophosphorylation activities of wild-type (*lane* 1) and mutant proteins (*lanes* 2–7) using 500 ng protein/reaction. Upper right panel: Autophosphorylation of H510A mutant protein with increasing concentrations (1X = 500 ng; 5X = 2.5 µg and 10X = 5 µg) of protein. Western blotting of mutant proteins was carried out using anti-His antibody (lower left and right panels). B. Far-UV CD spectra of mutant proteins. C. Gel filtration elution profile of wild-type (WT) and mutant (H510A and H510Q) proteins. V_o_ indicates void volume. I and II represent the molecular mass corresponding to dimeric and monomeric forms of mPPK1 respectively. Inset: Molecular mass calibration curve using different proteins. Notation used: F, Ferritin (440 kDa); C, Catalase (232 kDa); A, Aldolase (158 kDa); AD, Alcohol dehydrogenase (150 kDa); CA, Conalbumin (75 kDa) and BSA, Bovine serum albumin (66 kDa). Position of molecular mass markers is indicated.

PPK1s in prokaryotes are known to be histidine kinases. There are 22 His residues in mPPK1. To identify the phosphorylating His residue(s) of this protein, we mutated three conserved His residues (one at a time) to either Ala (H480A, H491A and H510A) or Gln (H480Q, H491Q and H510Q) and their autophosphorylating abilities (500 ng/reaction) were assessed. Among mutant proteins, H480A and H480Q did not affect the autophosphorylation activity (Fig. 1A, upper left panel, lanes 2 and 3). H491A and H491Q, on the other hand, did not show any phosphorylating abilities, suggesting predominant role of His-491 in the process (Fig. 1A upper left panel, lanes 4 and 5). Interestingly, H510A protein did not show any autophosphorylation, while H510Q had no effect on the activity (Fig. 1A upper left panel, lanes 6 and 7). However, use of ∼ 5-fold or more of H510A protein exhibited autophosphorylation indicating it to be a deficient mutant (Fig. 1A, upper right panel). Expression of mutant proteins was ensured in Western blotting with anti-His antibody (Fig. 1A, lower panels). Contrary to an earlier observation, wherein His residues of mPPK1 at position 491 and 510 were shown to be involved in autophosphorylation [27], we did not observe such role of His-510 *per se*. In fact, our results argue that the effect of mutation at His-510 of mPPK1 is rather amino acid specific.

To account for the discrepancy in the behavior of H510A and H510Q mutant proteins, we monitored their far-UV CD spectra. Compared to wild-type, we did not observe any appreciable alteration in far-UV CD spectra of the mutant proteins (Fig. 1B). Thus, it seems that there was no gross alteration in the secondary structure of the mPPK1 protein as a result of mutations. In molecular sieving chromatography, besides void volume fraction (oligomeric population) the elution profile of mPPK1 exhibited one major (197±8 kDa, n = 4) and a minor (87±5 kDa, n = 4) peaks in our experimental conditions (Fig. 1C, left panel). Considering the molecular mass of mPPK1 as ∼86 kDa, the wild-type protein eluted at these peaks very likely represented monomeric (minor) and dimeric (major) population. Accounting for the area under the peak, in mPPK1 monomeric form was ∼28% of dimeric population ( = 100%). Compared to the wild-type, H510Q protein did not display any significant alteration (Fig. 1C, right panel; monomer was ∼50% of dimeric form). However, there was a drastic alteration in monomeric population (∼181% of dimeric population) in the elution pattern of H510A protein which was inactive. Thus, these results insinuate that depending on the amino acid substitution, His-510 may contribute towards the oligomerization of mPPK1.

### Involvement of His-510 of mPPK1 in poly-P and ATP syntheses

**Figure 2:**
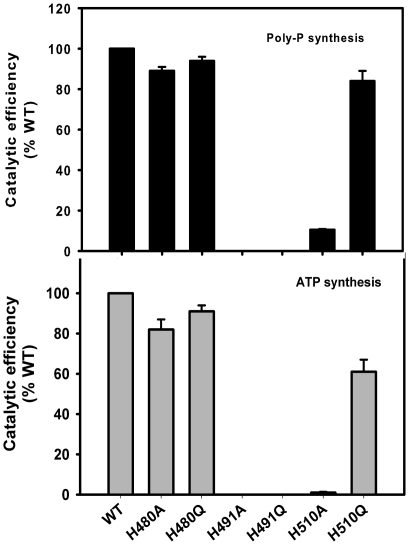
His-510 influences ATP synthesis ability of mPPK1. Effect of conserved His residue mutations on the kinetic efficiencies for poly-P synthesis and its mobilization (ATP synthesis) by mPPK1 was assessed (please see Tables 1 and 2). Kinetic efficiencies (*k*_cat_/*K*_m_) for poly-P and ATP syntheses were determined from each experiment and expressed as % wild-type control (100% poly-P synthesis  = 34.7±1.3 mM^−1^ s^−1^, while that of ATP synthesis  = 5743±646 mM^−1^ s^−1^).

In bacteria, PPK catalyzed reactions are known to be synthesizing poly-P from ATP and *vice-versa* [4]. Following incubation of mPPK1 with ATP, the reaction product was mixed with a basic dye Toluidine Blue O and poly-P formation (forward reaction) was determined (see ‘Materials and Methods’). The rate of poly-P synthesizing ability of mPPK1 as a function of increasing concentrations of ATP when plotted through a Michaelis-Menten curve yielded *K*_m_ and *k*_cat_ values of ∼1.35 mM and ∼ 47 s^−1^ respectively (Table 1). We further monitored the effect of His mutations on the poly-P synthesizing functions of mPPK1. As has been observed with the autophosphorylation activity, catalytic efficiency of the mPPK1 for the poly-P synthesis was almost or very close to that of the wild-type in H480A, H480Q and H510Q (Fig. 2, upper panel and also see Table 1). H491A or H491Q did not show any activity at all. H510A, on the other hand, exhibited a compromised behavior (Fig. 2, upper panel). This was anticipated since H510A mutant protein was deficient for autophosphorylation ability, which precedes poly-P synthesizing activity (see Fig. 1A). Thus, our results unequivocally established that His-491 is crucial for the mPPK1 mediated poly-P synthesis.

**Table 1:** Kinetics for poly-P and ATP synthesizing activities by PPK1 mutants.

	Poly-P synthesis			ATP synthesis		
Constructs	*K_m_*	*k*cat	*k*cat/*K_m_*	*K_m_*	*k*cat	*k*cat/*K_m_*
	*mM*	*s^−1^*	*mM ^−1^ s ^−1^*	*mM*	*s^−1^*	*mM ^−1^ s ^−1^*
WT	1.35±0.05	46.9±0.1	34.7±1.3	0.16±0.024	943±12	5743±646
H480A	1.47±0.02	45.6±1	31.0±0.6	0.20±0.003	950±2	4690±71
H480Q	1.38±0.012	45±0.6	32.6±0.8	0.18±0.004	971±14	5222±147
H491A	ND^a^	ND^a^	ND^a^	ND^a^	ND^a^	ND^a^
H491Q	ND^a^	ND^a^	ND^a^	ND^a^	ND^a^	ND^a^
H510A	5.2±0.14	55.4±1.2	10.5±0.4	6.8±0.4	332±65	49±12
H510Q	1.52±0.02	44.6±1.3	29.0±0.8	0.176±0.01	622±11	3529±171
Y524A	13.0±0.7	55.0±3.5	4.2±0.4	2±0.1	994±5	998±33

Poly-P synthesizing activity was assessed with mPPK1 or different mutants (40 µg/reaction) by using ATP (2–20 mM for Y524A and 0.125–10 mM for others). ATP synthesis activity was monitored by incubating purified mPPK1 or mutants (5 µg/reaction) with different concentrations of ADP (1–12 mM for H510A and 12.5–800 µM for others) in the presence of poly-P20. *K*_m_ and *V*_max_ values in each case were determined by non-linear regression analysis of Michaelis-Menten equation. *k*_cat_ values were calculated considering molecular mass of the recombinant mPPK1 as 86 kDa. Results are presented as Mean ± SD (n = 7 for wild-type and 3 for others).

a not detectable.

In determining the role of conserved His residue mutations on the ATP synthesizing activity of mPPK1 (reverse reaction), an enzyme-coupled assay system was employed where commercially available poly-P20 was used as phosphate donor (see ‘Materials and Methods’). Kinetics of ATP synthesis was assessed following incubation of poly-P20 with mPPK1 in the presence of ADP and subsequent spectrophotometric monitoring of conversion of NAD to NADH. Plotting of velocity of reaction as the function of ADP concentration yielded a typical Michaelis-Menten curve for ATP synthesized by mPPK1 and parameters were calculated from the non-linear regression analysis. The *K*_m_ and *k*_cat_ values for ATP synthesis by mPPK1 from ADP were ∼0.16 mM and ∼943 sec^−1^ respectively (Table 1). Like mPPK1 mediated forward reaction, *k*_cat_/*K*_m_ values for ATP synthesis were unaffected with H480A/H480Q and it is seriously compromised with the use of H491A/H491Q mutant proteins (Fig. 2, lower panel). Both H510A and H510Q proteins, on the other hand, displayed a significant decrease in catalytic efficiency compared to the wild-type for ATP synthesis (Fig. 2, lower panel and also see Table 1). Thus, it is apparent that His-510 in addition to His-491, contributed in the reverse reaction mediated by mPPK1.

### C-terminal domain residues of mPPK1 affecting poly-P synthesis also affect ATP synthesizing ability

**Figure 3:**
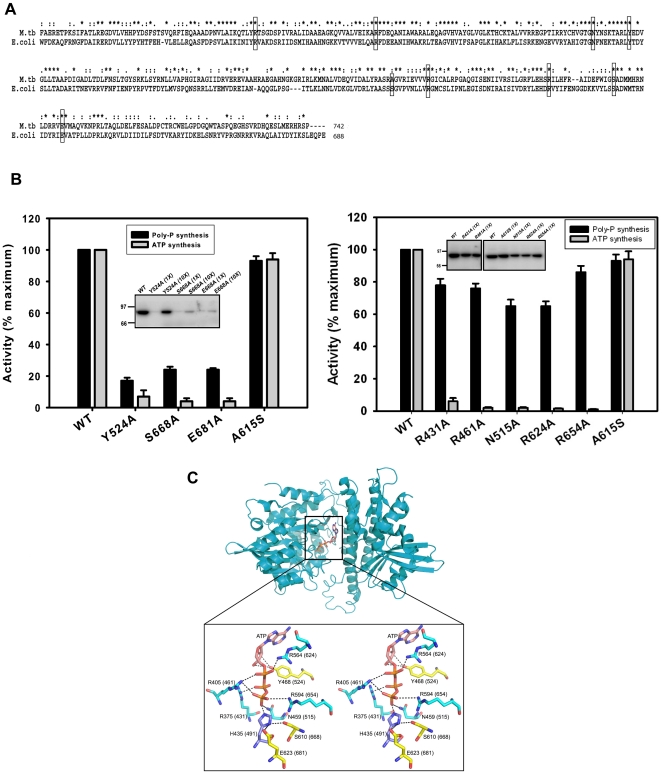
Amino acid residues involved in poly-P and ATP syntheses are distinct. A. Alignment of amino acid residues from *M. tuberculosis* (M.tb; residues 378–742) and *E. coli* (E. coli; residues 322–687). Residues selected for mutations are highlighted in box. B. Effect of mutations on poly-P and ATP synthesizing activities of mPPK1. Mutations affecting both the activities (left panel) and only ATP synthesis (right panel). The amino acid Ala-615 which is away from active site was chosen as a control. Activities in each case are expressed as % wild-type control. *Inset*: Autophosphorylation profile of different C1 and C2 domain mutants (Notations used: 1X = 500 ng protein/reaction and 10X = 5 µg protein/reaction). C. The PPK1 from *E. coli* (PDB ID: 1XDP) showing the conserved C- terminal domain residues chosen for the mutational analysis. The carbon residues shown in yellow represent the mutation affecting both poly-P and ATP syntheses while cyan represents the mutation affecting only ATP synthesis. The conserved phospho-histidine (His-491) residue is shown in purple (carbon atom). The mPPK1 residue numbers are shown in parentheses.

Residues of the C-terminal C1/C2 domains of bacterial PPKs are highly conserved and crucial for the catalytic activity of the enzyme [18]. Comparison of amino acid sequences of C-terminal domains between *E. coli* and *M. tuberculosis* exhibited about 72% similarity (residues conserved among all characterized PPK1 from different organisms are shown in Fig. S2). Moreover, the residues involved in nucleotide binding and catalytic activity are essentially unaltered between *E. coli* and *M. tuberculosis* [Fig 3A]. Therefore, various point mutants (R431A, R461A, N515A, Y524A, R624A, R654A, S668A, and E681A) were generated to evaluate the poly-P as well as ATP synthesizing abilities of mPPK1. Interestingly, we noticed that the mutation in amino acid residues, such as Tyr-524, Ser-668 and Glu-681 affected both the activities (Fig. 3B, left panel) while, the residues like Arg-431, Arg-461, Asn-515, Arg-624 and Arg-654 disrupted only the NTP synthesizing ability of mPPK1 (Fig. 3B, right panel). Analysis of these mutations in *E. coli* PPK1 structure revealed that the residues affecting both the activities are interacting either with sugar moiety of ATP (Y524A) or with His-491 (S668A and E681A) residue (Fig. 3C). Kinetic studies with Y524A mutant protein clearly showed less binding affinity for ADP/ATP compared to that of the wild-type mPPK1 (Table 1). In fact, *in vitro* kinase assay also did not display any autophosphorylation activity of Y524A mutant with 500 ng protein as used in wild-type. However, 10-fold excess of protein exhibited autophosphorylating ability of this mutant protein reflecting it to be a deficient mutant (Fig. 3B, left panel, inset). On the other hand, the residues interacting with His-491 (crucial for the functionality of the enzyme; see Fig. 1) are likely to play a role for its (His-491) proper positioning and if perturbed may affect the enzyme activity.

Furthermore, analysis of *E. coli* PPK1 structure revealed that the mutants (R431A, R461A, N515A, R624A and R654A) interacting with phosphate moiety of ATP (Fig. 3C). These interactions are very likely contributing to the proper orientation of phosphate moiety of ATP. Alteration in any such interactions would severely perturb the ADP binding (γ-phosphate moiety is absent) but would not affect the ATP binding to mPPK1. Therefore, these mutations are very unlikely to have any effect on poly-P synthesis (forward reaction) but definitely affect NTP synthesis (reverse reaction). Thus, it is apparent from our results that amino acids involved in NTP and poly-P syntheses are distinct in mPPK1.

### mPPK1 exhibits Auxiliary NDK activity

**Figure 4:**
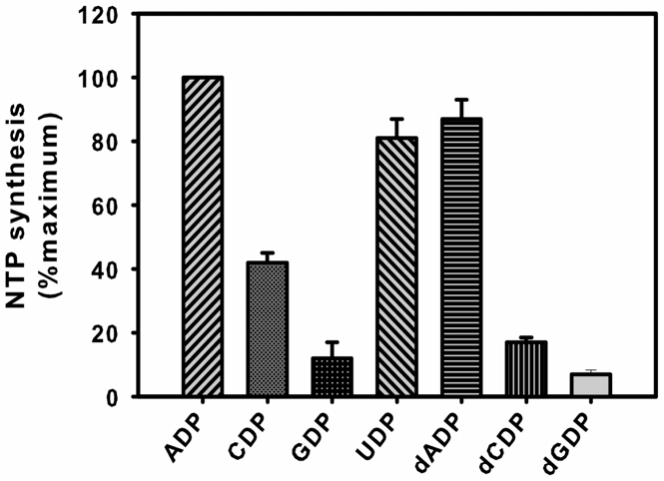
mPPK1 exhibits auxiliary NDK activity. NTP synthesizing ability of mPPK1 was evaluated by incubating purified protein (5 µg) with 5 mM of NDP or dNDPs in a reaction mix containing poly-P20 as phosphate donor. Amount of different NTPs/dNTPs formed/min/mg protein were calculated from standard curves as indicated under ‘Materials and Methods. The activity is expressed as % maximum (100% = 197±14 µmol/min/mg of protein, n = 3).

Nucleoside diphosphate kinase (NDK) in *M. tuberculosis* has already been shown to be responsible for maintaining nucleotide pools [31]. Available literature also indicated that excessive need of NTPs under stressed conditions are often met by poly-P by generating ATP through PPK1 or PPK2 mediated reverse reactions [9], [13], [32]. To gain insight on this aspect, NTP synthesizing ability of mPPK1 from poly-P was assessed by using different NDPs. As shown in Fig. 4, mPPK1 was able to synthesize ATP, dATP or UTP from poly-P20 with almost equal efficiency from different NDPs used. Interestingly, when compared ATP and GTP synthesizing abilities of mPPK1, affinity for the ADP as a substrate was ∼70 fold high as opposed to GDP (Table 2). We further compared the NTP synthesizing ability of PPK1 and PPK2 from *M. tuberculosis*. It was evident that mPPK2 was able to synthesize only ATP in our experimental conditions, albeit with less efficiency compared to that of mPPK1 (Table 2). Thus, our results highlighted the ability of mPPK1 to mimic NDK activity.

**Table 2:** Kinetics for NTP synthesizing activities by PPK1 and PPK2 from *M. tuberculosis*.

Enzyme	Substrate	*K_m_*	*k*cat	*k*cat/*K_m_*	No. of experiments
PPK1		*mM*	*s^−1^*	*mM ^−1^ s ^−1^*	
	ADP	0.16±0.024	943±12	5743±646	3
	GDP	11.3±0.6	645±3	57±3	3
PPK2					
	ADP	8±0.95	210±7	25.5±2	3

NTP synthesis assays were carried out following incubation of purified protein (5 µg/reaction for each protein) with 250 µM poly-P20 and different concentrations of ADP (12.5–800 µM for mPPK1 and 1–40 mM for mPPK2) or GDP (1–40 mM). *K*_m_ and *V*_max_ values were determined from non-linear regression analysis of Michaelis-Menten equation. For calculating *k*_cat_ values, molecular masses of the recombinant mPPK1 and mPPK2 are considered as 86 kDa and 36 kDa respectively. Results are presented as Mean ± SD.

## Discussion

The role of poly-P, the linear chains of several ortho-phosphate residues, in bacterial stress response is well-known [2], [3], [27], [33]. The principal enzyme responsible for its synthesis is polyphosphate kinase. Interestingly, the same enzyme is also involved in the mobilization of poly-P to NTP. Therefore, PPKs are among the crucial bacterial enzymes importance of which is beyond any doubt, especially in pathogens where its association in production of virulence factors has often been led to its consideration as a drug target [34]. In this context, we have focused on *M. tuberculosis*, where two genes encoding such polyphosphate kinases, *ppk1* and *ppk2*, are reported [14]. While the intra-cellular life of *M. tuberculosis* within macrophages has been shown to be regulated by mPPK1, mPPK2 acted synergistically with NDK in maintaining intracellular NTP pools under stressed conditions [27], [28]. In fact, DNA-based aptamer(s) for PPK2 have also been developed that inhibited the catalytic activity of the enzyme [35]. However, role of different amino acid residues of *M. tuberculosis* PPKs towards the functionality of the enzyme(s) has not yet been elucidated in detail. In this study, we concentrated on mPPK1 and carried out structure-function analysis of this enzyme to evaluate the contribution of different amino acid residues in poly-P synthesis as well as its mobilization.

The poly-P synthesis mediated by PPK1 involves sequential steps. These are NTP-binding to PPK1 as well as autophosphorylation of the protein followed by poly-P biosynthesis. We performed step-wise analysis of these events with mPPK1. Expectedly, His-tagged mPPK1 protein used in this study exhibited Mg^2+^/Mn^2+^ dependent autophosphorylation activity (Fig. S1B). Available literature indicated that both His-491 and His-510 are involved in autophosphorylation of mPPK1 [27]. To have an insight on this aspect, both the His residues at 491 and 510 positions were substituted with either Ala or Gln. Expectedly, none of the His-491 mutant proteins (H491A and H491Q) exhibited any autophosphorylation activity (Fig. 1A). Similarly, in autophosphorylation assay using same amount of protein as has been used for wild-type did not yield any activity for H510A. On the other hand, H510Q mutant protein displayed autophosphorylation, which is quite comparable to that of the wild-type (Fig. 1A). The autophosphorylation of the protein is followed by its poly-P synthesis ability and it is the last step of the forward reaction mediated by the mPPK1 [18]. Since these activities are inter-linked, we observed that the mutations of mPPK1 compromised for autophosphorylation also reflected in its poly-P synthesizing ability. Thus, H510A mutant protein was not an exception. In fact, H510A mutant protein showed an increased *K*_m_ value for ATP (∼3.8 fold) compared to that of the wild-type, which suggested that decreased affinity for the substrate presumably affected the catalytic efficiency of the enzyme for the poly-P synthesis (Fig. 2, upper panel and see Table 1). The difference in the behavior of H510A and H510Q mutant proteins is very likely indicative of the fact that His-510 *per se* have no contribution in poly-P synthesizing ability of mPPK1, but mutations depending on the amino acid substitution(s) affected the activity of the protein. On the other hand, His-491 is the phospho-histidine residue of this kinase, which is crucial for the functionality (both autophosphorylation and poly-P synthesis) of the mPPK1 (Figs. 1A and 2, upper panel).

PPK1 is also known to catalyze the ATP synthesis from poly-P as a part of its reverse reaction [11], [17]. We further monitored the behavior of these His residue mutants on the ATP synthesizing ability of mPPK1 using poly-P20 as the phosphate donor. Like poly-P synthesis, H491A or H491Q proteins hardly depicted any ATP producing capability (Fig. 2, lower panel and Table 1). Surprisingly, H510Q protein in addition to H510A displayed a significant decrease in catalytic efficiency compared to that of the wild-type for ATP synthesis. Kinetic analysis of the enzymatic activity of H510A protein revealed that there was ∼42.5 fold increase in *K*_m_ and ∼ 2.8 fold decrease in *k*_cat_ values compared to that of the wild-type, which resulted in overall ∼117 fold decrease in catalytic efficiency of the mPPK1 enzyme for ATP synthesis (Fig. 2, lower panel and Table 1). Unlike poly-P synthesis activity of mPPK1, catalytic efficiency of the H510Q enzyme for ATP synthesis was reduced by ∼1.6 fold compared to that of the wild-type and obviously this has happened due to decreased *k*_cat_ value (Table 1). Thus, our results for the first time ascertained that His-510 in addition to His-491 contributes in reverse reaction mediated by mPPK1 to generate ATP.

In our experimental set up the elution profile of mPPK1 in molecular sieving chromatography revealed two peaks corresponding to monomeric and dimeric populations of the protein. As expected, eluted samples from both the peaks when resolved in SDS-PAGE exhibited single band corresponding to its monomeric molecular mass and they were recognized by anti-mPPK1 antibody. However, in autophosphorylation assay we found that only dimeric (but not the monomeric) population was active (data not shown). This observation corroborated well with earlier reports that mPPK1 is active as a dimer [27]. Interestingly, in case of H510A protein, monomeric population was significantly increased compared to that of the wild-type (Fig. 1C, compare left and right panels). Although it is not clear from our study how His-510 is involved in this process *per se*, we speculate that the mutation has changed the tertiary structure which led to perturbation in the oligomerization of mPPK1. Further work in this direction is necessary to elucidate the mechanistic detail. Nonetheless, it is apparent from our results that His-510 of mPPK1 is critical for oligomerization, which has never been reported for any PPK1.

We further focused into the residues of the carboxy-terminal domain to have an insight on their roles towards the functionality of mPPK1. Among the PPK1s characterized to date, X-ray crystal structure of *E. coli* protein has already been determined [18]. In fact, both *E. coli* and *M. tuberculosis* PPK1 amino acid sequences exhibited considerable similarity (Fig. 3A). Therefore, *E. coli* PPK1 structure was used for designing different mutants of mPPK1 and the effect of mutations on poly-P as well as ATP synthesizing activities of the protein were monitored. For this, alanine scanning mutagenesis was performed through the C1 and C2 domains of mPPK1. As expected, we noticed that the mutations of amino acids involved in either binding of adenine/ribose moieties of ATP or interacting with phopho-His (His491) residue affected both the activities (Fig. 3B, left panel). On the other hand, mutation of residues responsible for proper positioning of phosphate group of ATP or ADP significantly disrupted only the NTP synthesizing function of mPPK1 (Fig. 3B, right panel). Interestingly, despite being conserved residues we observed that the mutation of Arg-431, Tyr-524 and Arg-624 of mPPK1 behaved differently than its *E. coli* counterpart [10]. This species specific behavior is not unusual and therefore demands structure determination of mPPK1 to unravel the mystery. However, it seems that the amino acids involved in poly-P and NTP syntheses reactions are distinct and well crafted for specific functions.

Nucleoside diphosphate kinase is a ubiquitous enzyme responsible for the maintenance of NTP pools in prokaryotes. It catalyzes the phosphorylation of various NDPs by utilizing NTPs as phosphate donor and thus generates different NTPs [36]. We have also characterized NDKs from intra cellular pathogens, like *M. tuberculosis* and *Salmonella typhimurium* [37], [38]. Since PPKs catalyze the ATP synthesis from poly-P as a part of reverse reaction, they are often been referred as auxiliary NDK [9]. Utilizing poly-P as phosphate donor, PPK1s in *E. coli* and *P. aeruginosa* are known to catalyze synthesis of different NTPs/dNTPs [9]. Our results revealed that mPPK1 synthesizes almost all NTPs and dNTPs (Fig. 4) with varied efficiency utilizing poly-P20 as phosphate donor (Table 2). At this juncture it is intriguing to mention that in certain microorganisms, PPK2 has been assigned to perform auxiliary NDK function. Utilization of poly-P by mPPK2 for synthesis of ATP [35] or GTP [28] has also been reported. However, in our experimental conditions we observed that the mPPK2 can synthesize only ATP (Table 2), which is at par with the properties of single domain PPK2 as observed in other bacteria [15].

Finally, our results gleaning a wealth of useful information about the structure-activity relationship governing the forward and reverse reactions mediated by mPPK1, which unequivocally established that amino acid residues involved in poly-P or ATP synthesis are distinct. However, residues affecting poly-P synthesis would also abrogate the NTP generating ability of mPPK1, while the residues affecting NTP synthesis might not have any effect on poly-P synthesizing ability of the protein. Since PPK1 is much conserved throughout the phylogeny, it seems our observations have broader implications and not solely restricted to *M. tuberculosis* only.

## Materials and Methods

### Materials

Restriction/modifying enzymes were obtained from New England Biolabs. Ni-NTA resin (Qiagen), glutathione-sepharose (GE Healthcare), DNA/gel band purification kit, ECL Western blotting kit, protein molecular weight markers (GE Healthcare) and Herculase fusion DNA polymerase (Stratagene) were commercially available. All other fine chemicals including sodium triphosphates pentabasic, sodium hexametaphosphate, sodium phosphate glass, toluidine blue O, creatine phosphate, creatine kinase, glycerol-6-phosphate dehydrogenase, β-nicotiamide adenine dinucleotide sodium salt were procured from Sigma Chemical Company. Oligos used in this study were custom synthesized (IDT/Ocimum Biosolutions/Sigma). [γ-^32^P] ATP (3000-5000Ci/mmol) was purchased from Jonaki Laboratories, Board of Radiation and Isotope Technology, Hyderabad India.

### Gene isolation, cloning and generation of mutants

Genes encoding two polyphosphate kinases, *ppk1* (Rv 2984, accession number EF555553) and *ppk2* (Rv 3232c, accession number EF555554) of *M. tuberculosis* H37Rv (virulent strain) exhibited 100% identity at the nucleotide level with its avirulent strain (H37Ra). In this study, we therefore, isolated genomic DNA from *M. tuberculosis* avirulent strain H37Ra [39] and used as the template(s) for the PCR amplification of *ppk1* or *ppk2*. PCR primers (Table S1) were designed based on *M. tuberculosis* genome sequence incorporating restriction sites (NdeI at 5′ end and HindIII at 3′ end for ease in cloning) and reactions were carried out using ‘Herculase fusion DNA polymerase’ enzyme (denaturation at 98°C for 4 min, followed by 30 cycles amplification; each cycle comprising of 98°C for 20 sec, 61.2°C for 20 sec, 72°C for 1.5 min and finally extension at 72°C for 3 min). PCR amplified fragment(s) after restriction digestion was cloned in expression vector (pET28c) following standard protocol [40] to obtain pET-mPPK1 or pET-mPPK2. For the generation of point mutants of mPPK1 (H480A, H480Q, H491A, H491Q, H510A, H510Q, R431A, R461A, N515A, Y524A, A615S, R624A, R654A, S668A and E681A) overlap extension PCR method was employed [41]. For each mutation two external (CP3 and CP4) and two internal primers incorporating mutation (Table S1) were designed. This was followed by two sets of primary and one set of secondary PCR reactions. Final product(s) after gel elution were restriction digested with NdeI/HindIII and ligated to the corresponding sites of pET28c. All these constructs along with the wild-type were individually transformed in *E. coli* strain DH5α to build up DNA. All mutations were confirmed by nucleic acid sequencing using an automated DNA sequencer (Applied Biosystems).

### Expression and purification of recombinant proteins

The pET-mPPK1 or different mutant constructs were expressed following transformation individually in *E. coli* strain BL21(DE3). For pET-mPPK2 expression, plasmid DNA was co-transformed with chaperonic vector pkJE7 (Takara Bio Inc.) in the same host to obtain mPPK2 in solubilized form. For large scale preparation of different recombinant proteins BL21(DE3) cells harboring different constructs were grown overnight (14h at 37°C) in LB broth containing appropriate antibiotics (50 µg/ml kanamycin for pET-mPPK1 or mutant constructs while 50 µg/ml kanamycin and 20 µg/ml chloramphenicol for pET-mPPK2). Overnight cultures were re-inoculated (1% inoculum) in fresh LB broth supplemented either with only kanamycin (pET-mPPK1 and its mutants) or with L-arabinose (2 mg/ml) and kanamycin/chloramphenicol (pET-mPPK2) and grown till A_600_ of ∼0.6 at 37°C. This was followed by induction with either 0.2 mM IPTG for 16 h at 16°C (for mPPK1 and its mutants) or with 0.4 mM IPTG for 4 h at 25°C (for mPPK2). Cells were harvested and resuspended in lysis buffer, 50 mM HEPES-KOH, pH-7.5 containing either 100 mM NaCl and 5% glycerol (for mPPK1 and its mutants) or 50 mM NaCl (for mPPK2) along with protease inhibitor cocktail (1 mM phenyl methylsulfonyl fluoride, 1 µg/ml of pepstatin and 1 µg/ml of leupeptin). Following sonication (20 min at 4°C with alternate cycles of 10 sec ‘on’ and 10 sec ‘off’) supernatant fractions were collected (centrifugation at 17,600 x g for 30 min at 4°C), loaded onto Ni-NTA columns and washed (10 bed volumes) with lysis buffer containing 10 mM (for mPPK1 or its mutants) or 5 mM (for mPPK2) imidazole. Finally, proteins were eluted in elution buffer (lysis buffer containing 100 mM imidazole) and stored at −80°C until used. The protein concentrations were measured by Bradford method [42].

### Autophosphorylation

Autophosphorylating ability of mPPK1 or its mutants was assessed in an *in vitro* kinase assay by incubating (30 min at 25°C) purified proteins (25 ng to 5 µg/reaction) in 1x kinase buffer (50 mM HEPES-KOH, pH-7.5, 10 mM MgCl_2,_ 40 mM ammonium sulfate) in the presence of 2 µCi of [γ-^32^P]ATP [16]. To monitor the effect of divalent cations on the autophosphorylation activity of the enzyme, Mg^2+^ was replaced with different divalent cations (Ca^2+^, Co^2+^, Cu^2+^, Mn^2+^, Ni^2+^ or Zn^2+^) in kinase buffer. SDS gel loading buffer (30 mM Tris-HCl, pH 6.8, 5% glycerol, 2.5% 2-mercaptoethanol, 1% SDS and 0.01% Bromophenol Blue) was used to terminate autophosphorylation reaction in each case. Samples were resolved in 10% SDS PAGE, stained with Coomassie Brilliant Blue, dried in a gel drier (Biorad) at 70°C for 1 h and finally analyzed in a phosphoimaging device (Biorad/Fuji) as well as exposed to Kodak X-Omat/AR films for autoradiography.

### Poly-P synthesis

Poly-P synthesis ability of different samples (mPPK1 or its mutants) were assessed by incubating enzyme (40 µg protein/reaction) in assay buffer (50 mM HEPES-KOH, pH 7.5 containing 40 mM ammonium sulfate, 4 mM magnesium sulfate, 60 mM creatine phosphate and 50 µg of creatine kinase) with substrate (0.125–10 mM ATP; total reaction volume  = 250 µl) for 1 h at 37°C. This was followed by addition of Toluidine Blue O reagent (0.5 mg/ml TBO in 0.1 N acetic acid; amount used  = 750 µl/reaction) and subsequent monitoring of change in the ratio of the absorbance of the mixture at A_530_ and A_630_ [43]. The values obtained were corrected by subtracting the blank reading (all ingredients except enzyme). Standard curves were prepared with known concentrations (1–32 µM) of poly-P20 (number indicates Pi chain length) and the amount of poly-P synthesized was expressed as µmoles of poly-P produced as phosphates/min/mg protein.

### NTP synthesis

NTP synthesizing activity of mPPK1 and its mutants or mPPK2 was determined by using poly-P20. In fact, utilizing a standard Pi release assay [44] we found that both the enzymes were able to release maximum Pi from poly-P20 compared to that of poly-P3 or poly-P65. For the NTP synthesis assay, following incubation at 37°C for 1 h, the reaction cocktail containing 50 mM HEPES -KOH pH 7.5, 10 mM MgCl_2_, 12.5–800 µM of ADP or 1–30 mM of GDP, 250 µM poly-P20, 50 mM ammonium sulfate and 5 µg of enzyme (volume 100 µl/reaction) was mixed with assay mix (50 mM HEPES-KOH, pH-7.5, 10 mM NaCl, 4 units Hexokinase, 5 mM glucose, 1.5 mM MgCl_2_, 1 unit glucose-6-phosphate dehydrogenase and 1 mM NAD; volume 700 µl/reaction). NAD was added last to initiate the reaction and its reduction to NADH is measured at 340 nm. The values obtained were corrected by subtracting the blank readings. In experiments with single point assay 5 mM NDPs/dNDPs were used. Amount of NTP/dNTP formed is calculated from a calibration curve made using known amount of commercially available NTPs/dNTPs.

### Western Blotting

For Western blotting, purified proteins (1 µg/well) were resolved in 10% SDS PAGE and transferred at 120 mV for 1 h to nitrocellulose membrane (0.45 µm) using transfer buffer (48 mM Tris/HCl, 39 mM glycine, 0.037% SDS and 20% methanol) in a mini-transblot apparatus (Bio-rad). Membranes were stained with Ponceau S to ensure transfer and processed using anti-His monoclonal (GE Healthcare) or anti-mPPK1 polyclonal antiserum raised in rabbit following the procedure described elsewhere [45]. After incubation with secondary antibody (anti-mouse or anti-rabbit IgG), blots were developed with ECL detection system following the manufacturers (GE Healthcare) recommended protocol.

### Molecular sieving chromatography

Gel permeation chromatography of the Ni-NTA purified as well as dialyzed (to remove imidazole) His-tagged protein samples (mPPK1 and its mutant proteins prepared and eluted in 50 mM Tris-Cl, pH 7.5, 300 mM NaCl, 1 mM DTT and 10% glycerol; amount loaded/run  = ∼2 mg/ml) were performed using a Hiload™ 16/60 Superdex™ 200 prep grade column (flow rate of 1 ml/min) attached to an AKTA prime plus system.

### CD spectroscopy

CD spectra of different proteins (wild-type mPPK1 and its different mutants) were recorded in a Jasco J-810 spectropolarimeter. Measurements in the far-UV region (250–195 nm) were performed on protein solutions (0.15–0.2 mg/ml in 20 mM Tris-HCl, pH 7.5 containing 20 mM NaCl) using a cell with a pathlength of 0.2 cm at 25°C. The mean residue ellipticity (θ) was calculated using a mean residue molecular mass of amino acid as 110 Da. Each spectrum reported is an average of five scans. Blank spectra of aqueous buffer were used to correct the observed spectra.

### Bioinformatics and Structural analysis

The multiple sequence alignment was carried out by using Clustal W programme [46] with default input parameters. Crystal structure of *E. coli* PPK (PDB ID: 1XDP) was analyzed by displaying the structure in PYMOL [47] graphics program.

### Data analysis

Unless mentioned otherwise, all experiments in the present study are checked for their reproducibility at least three times and the results are represented as Mean ± S.D.

## Supporting Information

Figure S1 **Autophosphorylation activity of mPPK1.** **A.** Recombinant mPPK1 at different stages of purification. Overnight culture of BL21(DE3) cells transformed with pET-PPK1 were processed for purification as described in the ‘Materials and Methods’. A representative experiment showing mPPK1 samples after resolving in 10% SDS-PAGE was stained with Coomassie Brilliant Blue (upper panel) and Western blotting of the same with anti-His (middle panel) or anti-mPPK1 antibody (lower panel). *Lane* 1, molecular mass marker (LMW); *Lanes* 2–3, crude extract of cells harboring plasmid pET-mPPK1 with (induced) or without (uninduced) IPTG induction; *Lane* 4, Ni-NTA purified His-tagged mPPK1. Lane numbers are shown at the bottom. **B.** Autophosphorylation of mPPK1. Increasing concentrations (25 ng–5 µg) of purified mPPK1 protein was incubated with [γ-^32^P]-ATP in the presence of 10 mM Mg^2+^ and 40 mM ammonium sulphate. This was followed by separation of the reaction products by SDS-PAGE. The labeled proteins were visualized in a phosphoimaging device or by autoradiography of the dried gel (see ‘Materials and Methods’). Band intensities of the labeled proteins were determined using Scion Image software for windows. *Inset*, A representative autoradiograph of this experiment. **C.** Effect of divalent cations on autophosphorylation activities of mPPK1. Autophosphorylation reaction (500 ng/reaction) was carried out in presence (10 mM) or absence (None, *lane* 1) of different divalent cations (*lanes* 2–8) as indicated.
(TIF)

Figure S2 **Multiple sequence alignment of PPK1s characterized from different organisms.** Residues chosen for the mutation in C1 and C2 domains are conserved in all characterized PPK1 are highlighted in box.
(TIF)

Table S1 **List of PCR primers used in this study.**
(DOC)
