# Supplementary figures and images for: Amino Acids Involved in Polyphosphate Synthesis and Its Mobilization Are Distinct in Polyphosphate Kinase-1 from Mycobacterium tuberculosis
Source: PLoS One. 2011 Nov 14;6(11):e27398. doi: 10.1371/journal.pone.0027398 (PMC3215733; doi:10.1371/journal.pone.0027398)

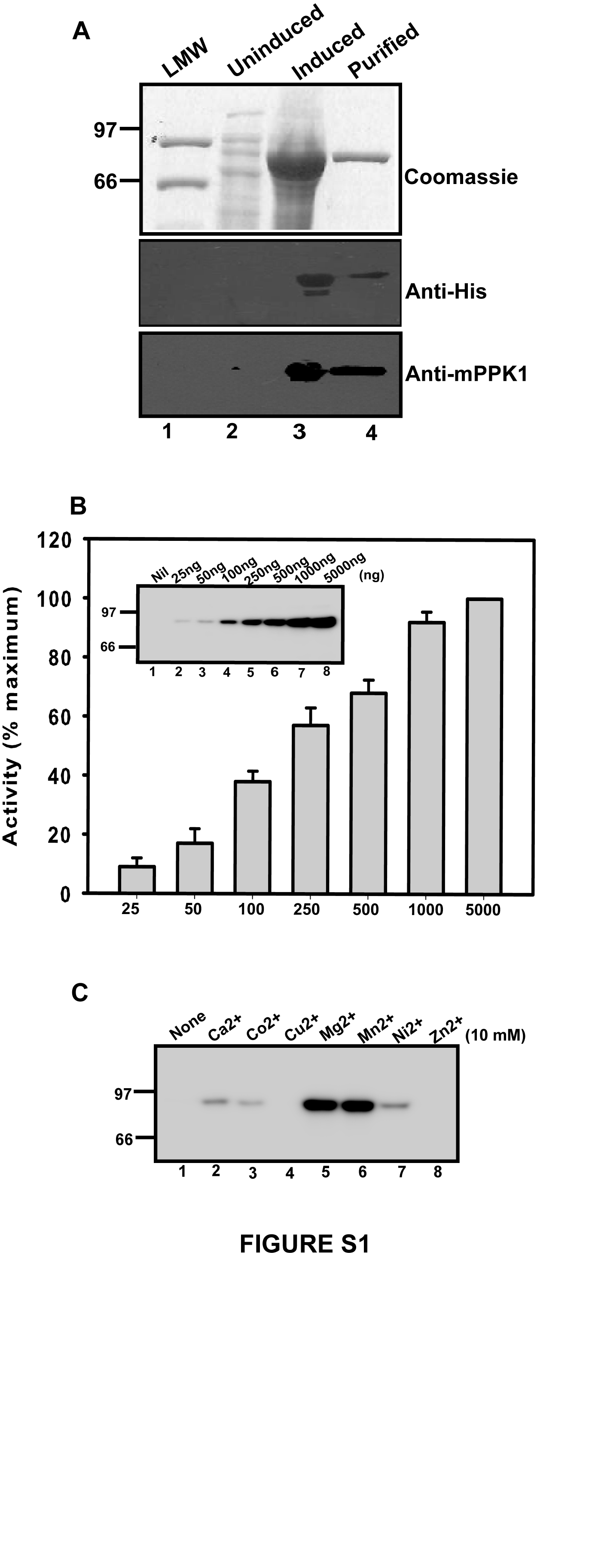

Supplement: Figure S1 — Autophosphorylation activity of mPPK1. A. Recombinant mPPK1 at different stages of purification. Overnight culture of BL21(DE3) cells transformed with pET-PPK1 were processed for purification as described in the ‘Materials and Methods’. A representative experiment showing mPPK1 samples after resolving in 10% SDS-PAGE was stained with Coomassie Brilliant Blue (upper panel) and Western blotting of the same with anti-His (middle panel) or anti-mPPK1 antibody (lower panel). Lane 1, molecular mass marker (LMW); Lanes 2–3, crude extract of cells harboring plasmid pET-mPPK1 with (induced) or without (uninduced) IPTG induction; Lane 4, Ni-NTA purified His-tagged mPPK1. Lane numbers are shown at the bottom. B. Autophosphorylation of mPPK1. Increasing concentrations (25 ng–5 µg) of purified mPPK1 protein was incubated with [γ-32P]-ATP in the presence of 10 mM Mg2+ and 40 mM ammonium sulphate. This was followed by separation of the reaction products by SDS-PAGE. The labeled proteins were visualized in a phosphoimaging device or by autoradiography of the dried gel (see ‘Materials and Methods’). Band intensities of the labeled proteins were determined using Scion Image software for windows. Inset, A representative autoradiograph of this experiment. C. Effect of divalent cations on autophosphorylation activities of mPPK1. Autophosphorylation reaction (500 ng/reaction) was carried out in presence (10 mM) or absence (None, lane 1) of different divalent cations (lanes 2–8) as indicated. (TIF) [file pone.0027398.s001.tif]

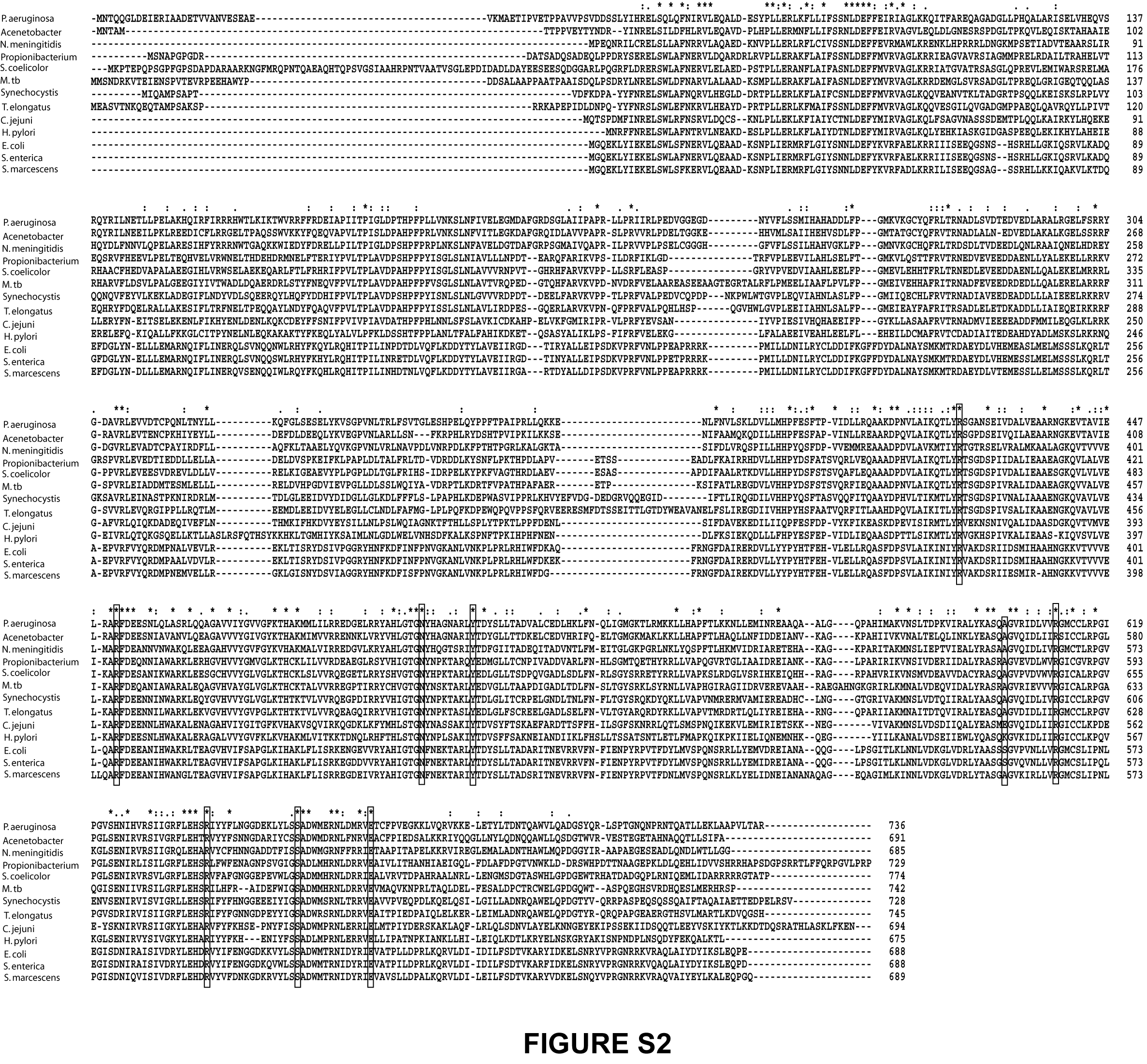

Supplement: Figure S2 — Multiple sequence alignment of PPK1s characterized from different organisms. Residues chosen for the mutation in C1 and C2 domains are conserved in all characterized PPK1 are highlighted in box. (TIF) [file pone.0027398.s002.tif]
