# Supplementary material for: Amino Acids Involved in Polyphosphate Synthesis and Its Mobilization Are Distinct in Polyphosphate Kinase-1 from Mycobacterium tuberculosis
Source: PLoS One. 2011 Nov 14;6(11):e27398. doi: 10.1371/journal.pone.0027398 (PMC3215733; doi:10.1371/journal.pone.0027398)
Supplement: Table S1 — List of PCR primers used in this study. (DOC) [file pone.0027398.s003.doc]

**Table S1. List of PCR primers used in this study.**

Primer code Sequence Gene

CP3 (sense) 5’-TTAAGTTCTAGACATATGATGAGCAATGAT-3’ **PPK1**

CP4 (antisense) 5’-AAATTAAGCTTTCAGGGGCTGCGGTG-3’

CP5 (sense) 5’-GTCGGACTCAAGACGGCCTGCAAGACC-3’ **H491A**

CP6 (antisense) 5’-GGTCTTGCAGGCCGTCTTGAGTCCGAC-3’

CP9 (sense) 5’-GGCGAGTCGCGGTTATGGCTCAAGTCA-3’ **E681A**

CP10 (antisense) 5’-TGACTTGAGCCATAACCGCGACTCGCC-3’

CP13 (sense) 5’-GAGTTCTGGATAGGCGCCGCCGACATGAT-3’ **S668A**

CP14 (antisense) 5’-ATCATGTCGGCGGCGCCTATCCAGAACTC-3’

CP15 (sense) 5’-GAGATCAAGGCAGCCTTCGACGAACAG-3’ **R461A**

CP16 (antisense) 5’-CTGTTCGTCGAAGGCTGCCTTGATCTC-3’

CP25 (sense) 5’-GTCGGCACCGGCGCTTACAACAGTAA-3’ **N515A**

CP26 (antisense) 5’-TTACTGTTGTAAGCGCCGGTGCCGAC-3’

CP27 (sense) 5’-AAGACAGCACGACTCGCCGAGGACG-3’ **Y524A**

CP28 (antisense) 5’-CGTCCTCGGCGAGTCGTGCTGTCTT-3’

CP29 (sense) 5’-GAGGTGGTGGTAGCCGGCATCTGCG-3’ **R624A**

CP30 (antisense) 5’-CGCAGATGCCGGCTACCACCACCTC-3’

CP32 (sense) 5’-CGAGCACTCGGCGATCCTCCATTTCC-3’ **R654A**

CP33 (antisense) 5’-GGAAATGGAGGATCGCCGAGTGCTCG-3’

CP42 (sense) 5’-CAGACGCTGTACGCCACCTCCGG-3’ **R431A**

CP43 (antisense) 5’-CCGGAGGTGGCGTACAGCGTCTG-3’

CP44 (sense) 5’-GGTACTGCGCTGTCGGAACCGGCAA-3’ **H510A**

CP45 (antisense) 5’-TTGCCGGTTCCGACAGCGCAGTACC-3’

CP46 (sense) 5’-GGTACTGTCAAGTCGGCACCGGCAATTA-3’ **H510Q**

CP47 (antisense) 5’-TAATTGCCGGTGCCGACTTGACAGTACC-3’

CP48 (sense) 5’-GCGTCGCGAAGCGGTGTGC-3’ **A615S**

CP49 (antisense) 5’-GCACACCGCTTCGCGACGC-3’

CP50 (sense) 5’-GCTCAAGACGCAATGCAAGACCGCCT-3’ **H491Q**

CP51 (antisense) 5’-AGGCGGTCTTGCATTGCGTCTTGAGC-3’

CP52 (sense) 5’-CGGGCTCAAGACAGCATGCAAGACCG-3’ **H480A**

CP53 (antisense) 5’-CGGTCTTGCATGCTGTCTTGAGCCCG-3’

CP 54 (sense) 5’-GAACAAGCAGGCGTGCAAGTGGCGTAC 3’ **H480Q**

CP 55 (antisense) 5’-GTACGCCACTTGCACGCCTGCTTGTTC 3’
